# Fluctuation Theorem for Information Thermodynamics of Quantum Correlated Systems

**DOI:** 10.3390/e25010165

**Published:** 2023-01-13

**Authors:** Jung Jun Park, Hyunchul Nha

**Affiliations:** 1 School of Computational Sciences, Korea Institute for Advanced Study, Seoul 02455, Republic of Korea; 2 Department of Physics, Texas A&M University at Qatar, Education City, Doha 23874, Qatar

**Keywords:** fluctuation theorem, quantum thermodynamics, quantum correlation, non-equilibrium, time-reversed process

## Abstract

We establish a fluctuation theorem for an open quantum bipartite system that explicitly manifests the role played by quantum correlation. Generally quantum correlations may substantially modify the universality of classical thermodynamic relations in composite systems. Our fluctuation theorem finds a non-equilibrium parameter of genuinely quantum nature that sheds light on the emerging quantum information thermodynamics. Specifically we show that the statistics of quantum correlation fluctuation obtained in a time-reversed process can provide a useful insight into addressing work and heat in the resulting thermodynamic evolution. We illustrate these quantum thermodynamic relations by two examples of quantum correlated systems.

## 1. Introduction

With current quantum technologies to control a few particles or quanta at microscopic level, much attention has been paid to understand how quantum principles play in the thermodynamics of quantum systems [1,2,3,4]. The quantum thermodynamics particularly far from equilibrium is an important field of study for open quantum systems under environmental interactions. It brings novel thermodynamic results by using various tools from both classical and quantum regimes. In the fluctuation dominant regime, thermodynamics may be addressed by a wide range of strategies to explain how the thermal fluctuations affect the evolution of system coupled to a thermal environment. One of the most remarkable achievements particularly in the non-equilibrium (NE) thermodynamics is the fluctuation theorem (FT) [5,6,7,8,9,10,11,12,13,14,15,16,17,18,19,20,21,22,23,24,25,26,27,28,29,30,31,32], which provides a central tool to reveal an interplay between the probabilistic behavior of entropy production and the second law of thermodynamics [33,34,35,36,37,38,39,40,41,42,43,44,45,46,47,48,49,50].

Motivated by Szilard’s seminal work that links information theory to thermodynamics [51,52], the so-called information thermodynamics has become a crucial framework to capture the role played by information in the emerging thermodynamics. One of its directions is to elucidate how the presence of correlations in a composite system can modify its thermodynamic evolution in and out of equilibrium in view of irreversibility measured by entropy production (EP) in NE processes [53,54,55,56,57,58,59,60,61,62]. In particular, the second law of thermodynamics as a universal principle may be derived from FT that is expressed as

(1) $$\langle e^{-\sigma +\Delta I}\rangle =1,$$

where σ is the entropy production (EP) and ΔI the change in the correlation of the composite system. This tells that the ensemble average of exponent incorporating EP for individual systems and a thermal bath plus a change of total correlation between the systems becomes unity independent of details that NE processes may have. The information FT was first demonstrated based on classical trajectory scenarios [53,54] and later extended to different scenarios in both classical and quantum domains [55,56,57,59,60,61,62].

For the case of quantum systems [9,10,17,63], the statistics of the fluctuating EP and the mutual information may be obtained by performing quantum measurements at the initial time and at the final time [55,56,57,59]. However, such quantum measurements destroy information on quantum correlation, thereby inappropriate to examine how quantum principles play in the emerging thermodynamic behaviors. Of course, quantum correlation may grow during the quantum dynamics after measurements, but it is a different correlation from the one that has existed in the initial quantum state before the system-reservoir interaction. Thus, one may instead use various quantum analyses to infer the effect of quantum fluctuations in terms of a quasi-probability [21] or quantum channel [19,20,29,30] to assess the quantum information-theoretic quantities.

In this paper, we aim at explicitly manifesting the contributions made by quantum fluctuation of correlation to the quantum thermodynamics of an open quantum system. Specifically we establish a detailed fluctuation theorem (DFT) in a form that enables us to elucidate those quantum contributions distinctly by observing their changes in the time-reversed process, while addressing classical contributions to EP in the usual time-forward manner. For this purpose, quantum probabilities in both of the time directions are introduced by reflecting on the quantum incompatibility, thereby obtaining a term representing genuine quantum nature of correlation. We show that our approach provides a very useful tool to understand quantum dissipative thermodynamics of correlation compared to the previously-known approaches and thermodynamic inequalities. We illustrate our theorem by applying our approach to some non-equilibrium scenarios in view of heat and work for quantum correlated systems highlighting our advantages over the known results.

## 2. Fluctuation Theorem for Open Bipartite Quantum System

We intend to generalize the classical FT in Equation (1) to quantum domains by incorporating quantum correlations explicitly. We start by considering a non-equilibrium process for a bipartite quantum system $\rho_{AB}\in H_{A}⊗H_{B}$ composed of two subsystems *A* and *B* with $\rho_{A}\in H_{A}$ and $\rho_{B}\in H_{B}$, each interacting with a thermal reservoir $\rho_{R}\;\in \;H_{R}$. An arbitrary quantum bipartite state $\rho_{AB}^{i}$ initially decoupled from $\rho_{R}^{i}$ evolves into a final state $\rho_{ABR}^{f}\;=\;U\rho_{AB}^{i}⊗\rho_{R}^{i}U^{†}$, where the process is described by a unitary operator *U*. The density operators of the joint system and the subsystems are initially given by $\rho_{AB}^{i}\;=\;\sum_{m}p_{m}\left|m\rangle \langle m\right|$ and $\rho_{A(B)}^{i}\;=\;{\mathrm{Tr}}_{B(A)}\left(\rho_{AB}^{i}\right)\;=\;\sum_{a(b)}p_{a(b)}\left|a\left(b\right)\rangle \langle a\left(b\right)\right|$, respectively, in the eigen-state decomposition. The thermal reservoir is also described as $\rho_{R}^{i}=\sum_{r}p_{r}\left|r\rangle \langle r\right|$. After the nonequilibrium process, we denote the final states as $\rho_{AB}^{f}\;=\;\sum_{m^{\prime }}p_{m^{\prime }}|m^{\prime }\rangle \langle m^{\prime }|$ of the bipartite system, $\rho_{A}^{f}\;=\;\sum_{a^{\prime }}p_{a^{\prime }}|a^{\prime }\rangle \langle a^{\prime }|$ of subsystem *A*, $\rho_{B}^{f}=\sum_{b^{\prime }}p_{b^{\prime }}|b^{\prime }\rangle \langle b^{\prime }|$ of subsystem *B*, and $\rho_{R}^{f}=\sum_{r^{\prime }}p_{r^{\prime }}|r^{\prime }\rangle \langle r^{\prime }|$ of the thermal bath.

As mentioned before, using only classical joint probabilities to look into the information-thermodynamic quantities is not adequate for quantum thermodynamics [21,22]. Thus, in order to assess quantum fluctuations appropriately, we introduce a quantum joint probability reflecting the incompatibility between the state of the joint system and the subsystems [64,65,66,67]. To begin with, the probability for the system AB and the reservoir *R* to be found in |m〉 and |r〉 at $t_{i}$, and $|m^{\prime }\rangle$ and $|r^{\prime }\rangle$ at $t_{f}$ is given by

(2) $$p_{m,m^{\prime };r,r^{\prime }}\;=\;|\langle m^{\prime },r^{\prime }{\left|U|m,r\rangle \right|}^{2}p_{m}p_{r}.$$

In the above equation, $p_{m}p_{r}$ is the joint probability for the system and the reservoir to be in the states |m〉 and |r〉, respectively, at the initial time. On the other hand, $|\langle m^{\prime },r^{\prime }{\left|U|m,r\rangle \right|}^{2}$ represents a conditional probability of finding the system and the reservoir in the states $|m^{\prime }\rangle$ and $|r^{\prime }\rangle$, respectively, at the final time through the unitary dynamics *U* conditioned on the initial states |m,r〉, which makes sense of $p_{m,m^{\prime };r,r^{\prime }}$ as required. We then multiply the conditional probabilities $p_{a,b|m}$ and $p_{a^{\prime },b^{\prime }|m^{\prime }}$ referring to the case that the subsystem *A* and *B* are found in the states |a〉 ($|a^{\prime }\rangle$) and |b〉 ($|b^{\prime }\rangle$) upon the condition that the composite system AB and the reservoir *R* are in the states |m〉 ($|m^{\prime }\rangle$) and |r〉 ($|r^{\prime }\rangle$) at $t_{i}$ ($t_{f}$). This makes it possible to define a joint probability as

(3) $$p_{m,a,b,m^{\prime },a^{\prime },b^{\prime };r,r^{\prime }}\;=\;p_{m,m^{\prime };r,r^{\prime }}{\left|\langle m|a,b\rangle \right|}^{2}{\left|\langle m^{\prime }|a^{\prime },b^{\prime }\rangle \right|}^{2},$$

which is a quantum analogue of classical joint probabilities. By defining the quantum joint probability in this manner, quantum fluctuation of information on all of the joint system and the subsystems is kept intact as we do not perform quantum measurements that would otherwise destroy quantum correlations: Equation (3) is the representation of information *inferred* from the density operator of the bipartite system, not by direct measurements incurring disturbance onto the quantum system. To see its validity as probability, we sum $p_{m,a,b,m^{\prime },a^{\prime },b^{\prime };r,r^{\prime }}$ over all local indices $a,b,a^{\prime },b^{\prime }$. Then, we find the required relation $\sum_{a,b,a^{\prime },b^{\prime }}p_{m,a,b,m^{\prime },a^{\prime },b^{\prime };r,r^{\prime }}\;=\;p_{m,m^{\prime };r,r^{\prime }}$. In the same way, we can verify the marginal probabilities $p_{m^{\prime },a^{\prime },b^{\prime };r^{\prime }}$, $p_{m}$, *etc*. Note that, as in all other time-local approaches, we may also extend our approach to multiple point measurements $p_{m_{0},m_{1},\cdots ,r_{0},r_{1},\cdots }\;=\;|\langle m_{0}|a_{0},b_{0}\rangle {|}^{2}{\left|\langle m_{1}|a_{1},b_{1}\rangle \right|}^{2}\ldots .$

Our main interest is to identify the role of genuinely quantum fluctuations in the emerging thermodynamics of quantum systems. To this aim, we first derive an information FT that is generalized from the classical theorem adopting the quantum probability in Equation (3). The resulting theorem turns out to be

(4) $${\langle e^{-\sigma +\Delta I}\rangle }_{q}\;=\;\kappa ,$$

where $\sigma :=\Delta s_{A}+\Delta s_{B}+\Delta s_{R}$ is the usual entropy production defined by a sum of the entropies of all subsystems and a thermal reservoir, with $\Delta s_{X}:\;=\;-\ln p_{x^{\prime }}-\left(-\ln p_{x}\right)$ (X=A,B,R). On the other hand, $\Delta I:=I_{f}-I_{i}$ is the change in the classical mutual information $I\left(a,b\right)=\ln\left[p_{a,b}/p_{a}p_{b}\right]$ defined by the classical joint probability $p_{a,b}=\langle a,b|\rho_{AB}|a,b\rangle$. Note that the entropies $s_{A}$, $s_{B}$ and $s_{R}$ defined above corresponds to the von Neumann entropy $S\left(\rho \right)\equiv -\mathrm{Tr}\left[\rho \ln\rho \right]$. This is because that they use the probabilities $p_{a(a^{\prime })},p_{b(b^{\prime })}$, and $p_{r(r^{\prime })}$ of the states $\rho_{A}^{i(f)}$, $\rho_{B}^{i(f)}$, and $\rho_{R}^{i(f)}$ in the eigen-state basis, respectively, as constructed before.

Most importantly, the factor κ represents the contribution by genuinely quantum correlation as we show explicitly below. In a classical limit where quantum correlation is not involved, this factor approaches κ=1 recovering the result in Equation (1). (See Equation (8) and around for the definition of κ that becomes unity if there does not exist quantum correlation, i.e., the case of α=0 and δ=0.) The factor κ thus implies the departure from the classical universality by reflecting pure quantum fluctuations.

### 2.1. Deriving the FT in Equation (4)

The NE factor κ is formulated through a time-reversed process $\tilde{U}$, which starts with an initial state that corresponds to the final state of the composite system in the forward process and a reinstated thermal bath at the same temperature *T*. This process $\tilde{U}$ evolves the initial time-reversed state ${\tilde{\rho }}_{ABR}^{i}\;=\;{\tilde{\rho }}_{AB}^{i}⊗{\tilde{\rho }}_{R}^{i}$ into ${\tilde{\rho }}_{ABR}^{f}$. Similar to the time-forward process, we construct the relevant states in the time-reversed process, i.e., the final state ${\tilde{\rho }}_{AB}^{f}\;=\;{\mathrm{Tr}}_{R}\left({\tilde{\rho }}_{ABR}^{f}\right)\;=\;\sum_{\tilde{m}}p_{\tilde{m}}|\tilde{m}\rangle \langle \tilde{m}|$ with the marginal states ${\tilde{\rho }}_{A(B)}^{f}\;=\;\sum_{\tilde{a}\left(\tilde{b}\right)}p_{\tilde{a}\left(\tilde{b}\right)}|\tilde{a}\left(\tilde{b}\right)\rangle \langle \tilde{a}\left(\tilde{b}\right)|$. The final state of the reservoir is similarly given by ${\tilde{\rho }}_{R}^{f}={\mathrm{Tr}}_{AB}\left({\tilde{\rho }}_{ABR}^{f}\right)$, with ${\tilde{\rho }}_{R}^{f}=\sum_{\tilde{r}}p_{\tilde{r}}|\tilde{r}\rangle \langle \tilde{r}|$.

Using a time-reversal operator Θ, the initial state ${\tilde{\rho }}_{AB}^{i}$ of the time-reversed process can be expressed in terms of the final state of the time-forward process. That is, ${\tilde{\rho }}_{AB}^{i}\;=\;\sum_{{\tilde{m}}^{\prime }}{\tilde{p}}_{m^{\prime }}|{\tilde{m}}^{\prime }\rangle \langle {\tilde{m}}^{\prime }|$, where $|{\tilde{m}}^{\prime }\rangle \;=\;\Theta |m^{\prime }\rangle$. The joint probability of the time-reversed process is then given by

(5) $${\tilde{p}}_{m^{\prime },a^{\prime },b^{\prime },m,a,b;r^{\prime },r}={\tilde{p}}_{m^{\prime },m;r^{\prime },r}|\langle m^{\prime }|a^{\prime },b^{\prime }\rangle {|}^{2}{\left|\langle m|a,b\rangle \right|}^{2},$$

where ${\tilde{p}}_{m^{\prime },m;r^{\prime },r}\;=\;|\langle \tilde{m},\tilde{r}|\tilde{U}|{\tilde{m}}^{\prime },{\tilde{r}}^{\prime }{\rangle |}^{2}{\tilde{p}}_{m^{\prime }}{\tilde{p}}_{r^{\prime }}$. Using the identity $\tilde{U}=\Theta U^{†}\Theta^{†}$ due to the microscopic reversibility [14], we also have $|\langle \tilde{m},\tilde{r}|\tilde{U}|{\tilde{m}}^{\prime },{\tilde{r}}^{\prime }{\rangle |}^{2}=\left|\langle m,r\right|\Theta^{†}\tilde{U}\Theta |m^{\prime },r^{\prime }{\rangle |}^{2}=\left|\langle m,r\right|U^{†}|m^{\prime },r^{\prime }{\rangle |}^{2}$.

#### 2.1.1. Detailed FT

As formulated in the Crooks theorem [8], the time-reversed probability can be linked to the time-forward probability for a quantum bipartite system in a detailed microscopic description. That is, by defining $\alpha_{i(f)}\;=\;-\ln{\left|\langle m^{\left(\prime \right)}|a^{\left(\prime \right)},b^{\left(\prime \right)}\rangle \right|}^{2}\;=\;\ln p_{m(m^{\prime })}-\ln p_{m\left(m^{\prime }\right),a\left(a^{\prime }\right),b\left(b^{\prime }\right)}$, we obtain

(6) $$\begin{array}{c} \begin{array}{cc} & p_{m,a,b,m^{\prime },a^{\prime },b^{\prime };r,r^{\prime }}\\ = & {\tilde{p}}_{m^{\prime },a^{\prime },b^{\prime },m,a,b;r^{\prime },r}e^{\Delta s_{A}}e^{\Delta s_{B}}e^{\Delta s_{R}}e^{-\Delta I}e^{-\Delta \delta }, \end{array} \end{array}$$

with technical details in Appendix A. Here $\Delta s_{A}:=\ln\frac{p_{a}}{{\tilde{p}}_{a^{\prime }}},\Delta s_{B}:=\ln\frac{p_{b}}{{\tilde{p}}_{b^{\prime }}}$ and $\Delta s_{R}:=\ln\frac{p_{r}}{{\tilde{p}}_{r^{\prime }}}$ are used to represent each systems’ entropy change. In addition, $\Delta I:=I_{f}-I_{i}\;=\;\ln\frac{{\tilde{p}}_{a^{\prime },b^{\prime }}}{{\tilde{p}}_{a^{\prime }}{\tilde{p}}_{b^{\prime }}}-\ln\frac{p_{a,b}}{p_{a}p_{b}}$, and $\Delta \delta :=\delta_{f}-\delta_{i}\;=\;\ln\frac{{\tilde{p}}_{m^{\prime }}}{{\tilde{p}}_{a^{\prime },b^{\prime }}}-\ln\frac{p_{m}}{p_{a,b}}$ are used to represent the change of quantum correlation.

The detailed fluctuation theorem is therefore given by

(7) $$\frac{{\tilde{p}}_{m^{\prime },a^{\prime },b^{\prime },m,a,b;r^{\prime },r}}{p_{m,a,b,m^{\prime },a^{\prime },b^{\prime };r,r^{\prime }}}\;=\;e^{-\sigma +\Delta I+\Delta \delta }.$$

Here $\Delta \delta =\delta_{f}-\delta_{i}$ is the change of a quantum fluctuation defined by $\delta_{i(f)}\;=\;\ln p_{m(m^{\prime })}-\ln p_{a\left(a^{\prime }\right),b\left(b^{\prime }\right)}$ representing the distinction between two probabilities: $p_{m}$ addresses the probability of finding the quantum system in a global state *m*. In contrast, $p_{a,b}$ addresses the probability of finding the quantum system in local states *a* and *b*, e.g., after quantum measurements. These probabilities become identical, and then the fluctuation disappears, when the quantum incompatibility does not hold, i.e., $[\Pi_{m},\Pi_{a,b}]\;=\;0$, where $\Pi_{m}\;=\;|m\rangle \langle m|$ and $\Pi_{a,b}\;=\;|a\rangle \langle a|⊗\left|b\rangle \langle b\right|$. The condition is indeed related to a type of the measure of quantum correlations [68] given by an amount of gap between total correlation $I_{Q}$ and classical correlation $I_{C}$. The average of the fluctuation becomes $\langle \delta \rangle \;=\;I_{Q}-I_{C}\;=\;S\left(\sum_{a,b}\Pi_{a,b}\rho_{AB}\Pi_{a,b}\right)-S\left(\rho_{AB}\right)$, where the von Neumann entropy of a bipartite system is $S\left(\rho_{AB}\right)\;=\;-\sum_{m}p_{m}\ln p_{m}$ and of its post-measured state $S\left(\sum_{a,b}\Pi_{a,b}\rho_{AB}\Pi_{a,b}\right)\;=\;-\sum_{a,b}p_{a,b}\ln p_{a,b}$.

#### 2.1.2. Integral FT

The detailed FT in Equation (7) can then be used to obtain the factor κ in the integral FT in Equation (4). After rearranging the terms in Equation (7) as $p_{m,a,b,m^{\prime },a^{\prime },b^{\prime };r,r^{\prime }}e^{-\sigma +\Delta I}={\tilde{p}}_{m^{\prime },a^{\prime },b^{\prime },m,a,b;r^{\prime },r}e^{-\Delta \delta }$ and summing both the sides of the relation over all possible indices, we find

(8) $${\langle e^{-\sigma +\Delta I}\rangle }_{q}\;=\;{\langle \kappa_{m,m^{\prime }}\rangle }_{\tilde{R}}=\kappa ,$$

where ${\langle ...\rangle }_{\tilde{R}}$ indicates an average by the time-reversed joint probability ${\tilde{p}}_{m^{\prime },m}$. Here, $\kappa_{m,m^{\prime }}\;=\;\sum_{a,a^{\prime },b,b^{\prime }}e^{-\left(\alpha +\Delta \delta \right)}$ involves two genuinely quantum fluctuations, Δδ and $\alpha \;=\;\alpha_{i}+\alpha_{f}$, with $\alpha_{i(f)}\;=\;-\ln{\left|\langle m^{\left(\prime \right)}|a^{\left(\prime \right)},b^{\left(\prime \right)}\rangle \right|}^{2}\;=\;\ln p_{m(m^{\prime })}-\ln p_{m\left(m^{\prime }\right),a\left(a^{\prime }\right),b\left(b^{\prime }\right)}$ in a similar context to δ. The three indexed joint probability $p_{m\left(m^{\prime }\right),a\left(a^{\prime }\right),b\left(b^{\prime }\right)}$ is related to a quantification of correlation through quantum measurements [67], which outputs probabilities of $p_{m}=\mathrm{Tr}\rho_{AB}\Pi_{m}$ and $p_{a,b}=\mathrm{Tr}\rho_{AB}\Pi_{a,b}$. The non-classicality of the factor κ can be demonstrated by showing that α=0 and δ=0 when $[\Pi_{m},\Pi_{a,b}]\;=\;0$ for all defined indices, i.e., $\rho_{AB}\;=\;\sum \Pi_{a,b}\rho_{AB}\Pi_{a,b}$.

One may find the physical implication of κ based on the fact that κ is evaluated by assessing α and δ. These two terms are motivated from the observation that a composite state |m〉 and the local state |a,b〉 are distinguished if there exists quantum correlation. For the case of α, it is defined as $-\ln{\left|\langle m|a,b\rangle \right|}^{2}$, which becomes zero if the global state |m〉 is a product state, i.e., |m〉=|a,b〉, thereby quantifying quantum correlation between two subsystems. A similar argument can also be given to δ defined as $\ln p_{m}-\ln p_{a.b}$.

### 2.2. Thermodynamic Inequalities for Heat Transfer and Work

It is worth noting that the time-reversed average of the informational component is a statistical technique firstly introduced in Ref. [54] to show the role of information in the Jarzynski’s equality using feedback controls. Our approach here elucidates the role of quantum fluctuations by identifying the factor κ that involves statistical terms of genuinely quantum nature. Our fluctuation theorem in Equation (8) can also lead to a novel thermodynamic inequality that can be more useful than other known inequalities. Using Jensen’s inequality we readily obtain a thermodynamic inequality from Equation (8) as

(9) 〈σ〉≥〈ΔI〉−lnκ.

By exploiting the link between entropy and other thermodynamic quantities, one may find thermodynamic applications of Equation (9) in various scenarios.

#### 2.2.1. Heat Transfer

First, we derive a thermodynamic inequality about heat transfer. Suppose the bath is initially in a thermal equilibrium state in both of the forward and the reversed processes described as $\rho_{R}^{i}=\sum_{r}p_{r}\left|r\rangle \langle r\right|$ with $p_{r}\;=\;e^{-\beta E_{r}^{R}}/Z_{\beta }$ and ${\tilde{\rho }}_{R}^{i}=\sum_{r^{\prime }}p_{r^{\prime }}|r^{\prime }\rangle \langle r^{\prime }|$ with $p_{r^{\prime }}\;=\;e^{-\beta E_{r^{\prime }}^{R}}/Z_{\beta }$, respectively. The inequality in (9) then leads to a thermodynamic inequality of heat transfer as

(10) $$\beta \langle Q\rangle \leq \langle \Delta s\rangle -\langle \Delta I\rangle +\ln\kappa ,$$

where $\langle \Delta s\rangle \;=\;\langle \Delta s_{A}\rangle +\langle \Delta s_{B}\rangle$ is a change in entropy of the subsystems and $\beta Q:=\ln{\tilde{p}}_{r^{\prime }}-\ln p_{r}\;=\;\beta \left[E_{r}^{R}-E_{r^{\prime }}^{R}\right]$ is a heat transferred from a thermal bath to system. Conventionally, the saturation condition of heat transfer occurs for the case of reversible processes. In contrast, our inequality in Equation (10) may be saturated during a dissipative process of a bipartite state, as illustrated later with an example.

#### 2.2.2. Work

Second, we derive a thermodynamic inequality about work. Suppose a quantum bipartite system starts in an equilibrium state at a temperature *T*. The fluctuation of work *w* is known to satisfy the Jarzynski equality (JE) for a non-equilibrium process $U_{AB}$ [7]. Denoting the initial and the final total Hamiltonians as $H_{i}\;=\;\sum_{m}E_{m}^{i}\left|m\rangle \langle m\right|$ and $H_{f}\;=\;\sum_{m^{\prime }}E_{m^{\prime }}^{f}|m^{\prime }\rangle \langle m^{\prime }|$, respectively, the JE is given by $\langle e^{-\beta (w-\Delta F_{AB})}\rangle \;=\;1$, where $w\;=\;E_{m^{\prime }}^{f}-E_{m}^{i}$ is thermodynamic work for an isolated system and $\Delta F_{AB}\;=\;F_{AB}^{f}-F_{AB}^{i}$ is a change of free energy $F_{AB}^{i(f)}\;=\;-k_{B}T\ln Z_{AB}^{i(f)}$ with $Z_{AB}^{i(f)}\;=\;\mathrm{Tr}\left(e^{-\beta H_{i(f)}}\right)$.

On the other hand, in our approach of addressing quantum fluctuations in the time-reversed protocol, we can establish a work FT as

(11) $$\langle e^{-\beta (w-\Delta f_{qc})}\rangle \;=\;\kappa ,$$

with $\Delta f_{qc}\;=\;f_{qc}^{f}-f_{qc}^{i}$ by introducing a quasi free-energy quantity as $f_{qc}^{i(f)}\;=\;E_{m(m^{\prime })}^{i(f)}+\beta^{-1}\ln p_{a\left(a^{\prime }\right),b\left(b^{\prime }\right)}^{i(f)}$. Its proof is given in Appendix B, by first deriving

(12) $$\begin{array}{c} p_{m,a,b,m^{\prime },a^{\prime },b^{\prime }}={\tilde{p}}_{m^{\prime },a^{\prime },b^{\prime },m,a,b}e^{\beta (w-\Delta f_{qc})-\Delta \delta }, \end{array}$$

with the quantum fluctuation $\delta_{i(f)}\;=\;\ln p_{m(m^{\prime })}-\ln p_{a\left(a^{\prime }\right),b\left(b^{\prime }\right)}$. This leads to

(13) $$p_{m,a,b,m^{\prime },a^{\prime },b^{\prime };r,r^{\prime }}e^{-\beta (w-\Delta f_{qc})}\;=\;{\tilde{p}}_{m^{\prime },a^{\prime },b^{\prime },m,a,b;r^{\prime },r}e^{-\Delta \delta },$$

and Equation (11) is derived in the same way as done with Equation (4), with $\kappa \;=\;\sum_{m,m^{\prime }}{\tilde{p}}_{m^{\prime },m}\sum_{a,a^{\prime },b,b^{\prime }}e^{-[\alpha +\Delta \delta ]}$.

We may show a relationship between the usual free energy and the quasi free energy as $F_{AB}^{i(f)}-f_{qc}^{i(f)}\;=\;\beta^{-1}\ln\frac{p_{m(m^{\prime })}^{eq}}{p_{a\left(a^{\prime }\right),b\left(b^{\prime }\right)}^{i(f)}}$. We thus recover $f_{qc}\;=\;F_{AB}$ when the quantum fluctuations are nullified, e.g., $p_{m}^{eq}\;=\;p_{a,b}$. While the total system in equlibrium is affected by thermal fluctuations, there exist quantum fluctuations due to the incompatibility between the state of the joint system and the subsystems, which are reflected through the quasi-free energy quantity representing a quantum feature.

Using Jensen’s inequality, the work FT also leads to a thermodynamic inequality as

(14) $$\langle w\rangle \geq \langle \Delta f_{qc}\rangle -\beta^{-1}\ln\kappa ,$$

where $\langle w\rangle$ is the work done on the joint system. It may be compared with the conventional second law for bipartite systems expressed by $\langle w\rangle -\Delta F_{AB}\geq 0$. By using the relationship between the quasi free energy and the conventional free energy, we recast Equation (14) into $\langle w\rangle -\Delta F_{AB}\geq \beta^{-1}\left\{\gamma -\ln\kappa \right\}$, where $\gamma \;=\;\langle -\ln\frac{p_{m^{\prime }}^{eq}}{p_{a^{\prime },b^{\prime }}^{f}}\rangle -\langle -\ln\frac{p_{m}^{eq}}{p_{a,b}^{i}}\rangle$, finding that the inequality integrates extra elements provided from the quantum fluctuations and the time-reversed statistics.

### 2.3. Determining κ in Experiment

We here discuss how one may determine the term $\kappa \;=\;\sum_{m^{\prime },m}{\tilde{p}}_{m^{\prime },m}\sum_{a,ba^{\prime },b^{\prime }}e^{-[\alpha +\Delta \delta ]}$ to experimentally test our FT and the accompanying inequalities. The quantum correlation term κ has two contributions, α and δ, both of which are intimately connected. That is, $\alpha =-\ln{\left|\langle m|a,b\rangle \right|}^{2}$ and $\delta \;=\;\ln p_{m}-\ln p_{a.b}$, in which the indices m,a and *b* refer to the composite state $\rho_{AB}\;=\;\sum_{m}p_{m}\left|m\rangle \langle m\right|$, and the local states $\rho_{A(B)}\;=\;{\mathrm{Tr}}_{B(A)}\left(\rho_{AB}\right)\;=\sum_{a(b)}p_{a(b)}\left|a\left(b\right)\rangle \langle a\left(b\right)\right|$, respectively, in the *eigen-state* decomposition, as previously defined.

#### 2.3.1. Obtaining α and δ

At the initial time $t_{i}$ and the final time $t_{f}$, we may perform a quantum state tomography to obtain the initial and the final joint state, $\rho_{AB}^{\left(i\right)}\;=\;\sum_{m}p_{m}\left|m\rangle \langle m\right|$ and $\rho_{AB}^{\left(f\right)}\;=\;\sum_{m^{\prime }}p_{m^{\prime }}|m^{\prime }\rangle \langle m^{\prime }|$, respectively. Knowing the joint state suffices to determine the reduced local states $\rho_{A(B)}^{\left(i,f\right)}$. As this tomography then resolves the probabilities $\left\{p_{m},p_{m^{\prime }}\right\}$ and all the component states $\{|m\rangle ,|m^{\prime }\rangle ,|a\rangle ,|a^{\prime }\rangle ,|b\rangle ,|b^{\prime }\rangle \}$, one can calculate $\alpha_{i}=-\ln{\left|\langle m|a,b\rangle \right|}^{2}$ and $\alpha_{f}=-\ln{\left|\langle m^{\prime }|a^{\prime },b^{\prime }\rangle \right|}^{2}$ to give $\alpha =\alpha_{i}+\alpha_{f}$. In addition, the δ term, $\ln p_{m}-\ln p_{a.b}$, can also be calculated using the identified joint state $\rho_{AB}$ as $p_{a,b}=\mathrm{Tr}\{\rho_{AB}|a\rangle \langle a|⊗|b\rangle \langle b|\}$ for both of the initial and the final state.

In the above approach, we have proposed that the two terms α and δ are determined through only the joint-state reconstruction without requiring the local measurements at the start and the end of the thermodynamic procedures.

For the initial state $\rho_{AB}^{\left(i\right)}\;=\;\sum_{m}p_{m}\left|m\rangle \langle m\right|$, one may just prepare the system in a state |m〉 each time with a net probability $p_{m}$. In this alternative approach, one needs to perform the tomography only for the final state $\rho_{AB}^{\left(f\right)}$, and the resolution of all the terms can go as decribed before.

#### 2.3.2. Obtaining the Transition Probabilities ${\tilde{p}}_{m^{\prime },m}$

To finally determine $\kappa \;=\;\sum_{m^{\prime },m}{\tilde{p}}_{m^{\prime },m}\sum_{a,ba^{\prime },b^{\prime }}e^{-[\alpha +\Delta \delta ]}$, we also need to measure the transition probabilities ${\tilde{p}}_{m^{\prime },m}$ in the time-reversed process. With the time-reversal operator Θ, the initial state ${\tilde{\rho }}_{AB}^{i}$ of the time-reversed process is prepared in terms of the final state of the time-forward process, as ${\tilde{\rho }}_{AB}^{i}\;=\;\sum_{{\tilde{m}}^{\prime }}{\tilde{p}}_{m^{\prime }}|{\tilde{m}}^{\prime }\rangle \langle {\tilde{m}}^{\prime }|$, where $|{\tilde{m}}^{\prime }\rangle \;=\;\Theta |m^{\prime }\rangle$. That is, we prepare the state $|{\tilde{m}}^{\prime }\rangle$ with a probability ${\tilde{p}}_{m^{\prime }}$ and then let the system evolve to reach a final state ${\tilde{\rho }}_{AB}^{m^{\prime },f}$ conditioned on the initial state $|{\tilde{m}}^{\prime }\rangle$.

By performing measurement in the basis of $|\tilde{m}\rangle \;=\;\Theta |m\rangle$ on the final state, we determine the transition probability ${\tilde{p}}_{m^{\prime },m}={\tilde{p}}_{m^{\prime }}{\tilde{p}}_{m|m^{\prime }}={\tilde{p}}_{m^{\prime }}\mathrm{Tr}\{{\tilde{\rho }}_{AB}^{m^{\prime },f}|\tilde{m}\rangle \langle \tilde{m}|\}$. Alternatively, we may just perform the tomography for the final state to find ${\tilde{\rho }}_{AB}^{m^{\prime },f}$ and then calculate the conditional probability ${\tilde{p}}_{m|m^{\prime }}=\mathrm{Tr}\{{\tilde{\rho }}_{AB}^{m^{\prime },f}|\tilde{m}\rangle \langle \tilde{m}|\}$ using the basis of $|\tilde{m}\rangle$.

## 3. Example

### 3.1. Local Isothermal Process for Composite Quantum System

We illustrate our derived inequalities of heat transfer and work manifesting their tightness with an example of an isothermal process on each subsystem in an initially quantum correlated state. we first introduce an isothermal process for a single qubit system and then apply it to each subsystem of a bipartite system both in a time-forward and in a time-reversed manner.

**Figure 1:**
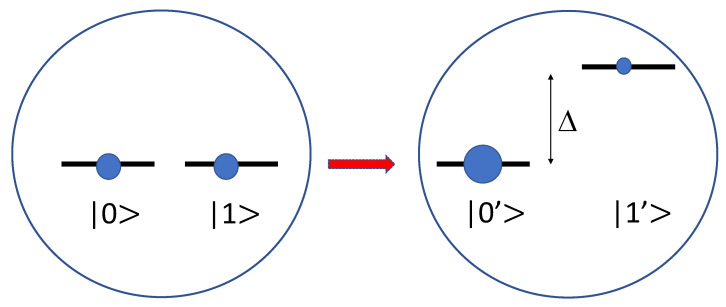
Two-level system undergoing an isothermal change of level splitting. The two states |0〉 and |1〉 that are initially degenerate are split into $|0^{\prime }\rangle$ and $|1^{\prime }\rangle$ with distinct energy levels by an amount Δ.

The isothermal process is performed by varying an energy gap Δ between a ground state |0〉 and an excited state |1〉 from 0 to Δ ($>>\beta^{-1}=k_{B}T$) in the qubit as shown in Figure 1. When we apply this isothermal process to each subsystem in a bipartite system, a degenerate state of the subsystem initially in a thermal equilibrium evolves from $\rho_{A(B)}^{i}⊗\rho_{R}\;=\;(\frac{1}{2}|a\left(b\right)=0\rangle \langle 0|+\frac{1}{2}|1\rangle \langle 1|)⊗\sum_{r}e^{-\beta E_{r}^{R}}/Z_{\beta }\left|r\rangle \langle r\right|$ to $\rho_{A(B)}^{f}⊗\rho_{R}\;=\;|0^{\prime }\rangle \langle 0^{\prime }|⊗\sum_{r}e^{-\beta E_{r}^{R}}/Z_{\beta }\left|r\rangle \langle r\right|$. The occupation probabilities in an equilibrium qubit state are given by $p_{0}\;=\;\frac{1}{1+e^{-\beta \Delta }}$ and $p_{1}=\frac{e^{-\beta \Delta }}{1+e^{-\beta \Delta }}$, which become $p_{0}\approx 1$ and $p_{1}\approx 0$ if the energy gap Δ is very large.

#### 3.1.1. Time-Forward Process

Let us now extend this isothermal process to each equilibrium subsystem of a maximally entangled bipartite system [69]. The joint system is initially in the state $\rho_{AB}^{i}⊗\rho_{R}^{A}⊗\rho_{R}^{B}\;=\;\sum_{m=0}^{3}p_{m}\left|m\rangle \langle m\right|⊗\sum_{r}e^{-\beta E_{r}^{R}}/Z_{\beta }\left|r\rangle \langle r\right|⊗\sum_{r^{\prime }}e^{-\beta E_{r^{\prime }}^{R}}/Z_{\beta }|r^{\prime }\rangle \langle r^{\prime }|$ in the Bell-state basis given by

(15) $$\begin{array}{cc} |0\rangle & =\frac{1}{\sqrt{2}}\left({|0\rangle }_{A}⊗{|0\rangle }_{B}+{|1\rangle }_{A}⊗{|1\rangle }_{B}\right),\\ |1\rangle & =\frac{1}{\sqrt{2}}\left({|0\rangle }_{A}⊗{|0\rangle }_{B}-{|1\rangle }_{A}⊗{|1\rangle }_{B}\right),\\ |2\rangle & =\frac{1}{\sqrt{2}}\left({|0\rangle }_{A}⊗{|1\rangle }_{B}+{|1\rangle }_{A}⊗{|0\rangle }_{B}\right),\\ |3\rangle & =\frac{1}{\sqrt{2}}\left({|0\rangle }_{A}⊗{|1\rangle }_{B}-{|1\rangle }_{A}⊗{|1\rangle }_{B}\right). \end{array}$$

After applying the aforementioned isothermal process to the bipartite system, the initial state |0〉 is changed to the final state $\rho_{AB}^{f}=|0^{\prime }\rangle \langle 0^{\prime }\left|\;=\;\right|0^{\prime }\rangle \langle 0^{\prime }{|}_{A}⊗|0^{\prime }\rangle \langle 0^{\prime }{|}_{B}$ in the eigenbases

(16) $$\begin{array}{cc} |0^{\prime }\rangle_{AB} & =|0^{\prime }\rangle_{A}⊗{|0^{\prime }\rangle }_{B},\\ |1^{\prime }\rangle_{AB} & =|0^{\prime }\rangle_{A}⊗{|1^{\prime }\rangle }_{B},\\ |2^{\prime }\rangle_{AB} & =|1^{\prime }\rangle_{A}⊗{|0^{\prime }\rangle }_{B},\\ |3^{\prime }\rangle_{AB} & =|1^{\prime }\rangle_{A}⊗{|1^{\prime }\rangle }_{B}. \end{array}$$

In this process, we evaluate the multi-indexed joint probabilities and the related contents in Appendix C.

#### 3.1.2. Time-Reversed Process

Similarly, we assess the joint probabilities in a time-reversed protocol by isothermally changing the energy gap from Δ to 0 in a time-reversed manner. During this process, the initial density operator ${\tilde{\rho }}_{AB}^{i}\;=\;|0^{\prime }\rangle \langle 0^{\prime }{|}^{AB}$ is changed to the density operator at the final time ${\tilde{\rho }}_{AB}^{f}\;=\;\frac{1}{2}{\left(|0\rangle \langle 0|+|1\rangle \langle 1|\right)}_{A}⊗\frac{1}{2}{\left(|0\rangle \langle 0|+|1\rangle \langle 1|\right)}_{B}$. We obtain the entropy and the information content for the time-reversed protocol in Appendix C.

### 3.2. Heat Transfer

We here demonstrate the thermodynamic inequality (10) of heat transfer with the above example of a dissipative, isothermal, process. Let us first compute the informational content lnκ, heat $\langle Q\rangle$, the entropy change of the subsystems $\langle \Delta s\rangle$, the classical mutual information $\langle \Delta I\rangle$, and then evaluate the validity and the saturation of the inequality.

Based on the definition of the time-reversed average, the quantum information κ is given by

(17) $$\begin{array}{cc} \kappa & =\sum_{m^{\prime },m}{\tilde{p}}_{m^{\prime },m}\sum_{a,ba^{\prime },b^{\prime }}e^{-[\alpha +\Delta \delta ]}\\ \; & ={\tilde{p}}_{0^{\prime },0}[e^{-[\alpha \left(0,0,0,0^{\prime },0^{\prime },0^{\prime }\right)+\Delta \delta \left(0,0,0,0^{\prime },0^{\prime },0^{\prime }\right)]}\\ \; & +e^{-[\alpha \left(0,1,1,0^{\prime },0^{\prime },0^{\prime }\right)+\Delta \delta \left(0,1,1,0^{\prime },0^{\prime },0^{\prime }\right)]}]\\ \; & =\frac{1}{4}\times \left[e^{0}+e^{0}\right]\;=\;\frac{1}{2}, \end{array}$$

where the component of the quantum incompatibility fluctuation is $\alpha \left(0,0,0,0^{\prime },0^{\prime },0^{\prime }\right)\;=\alpha_{i}\left(0,0,0\right)+\alpha_{f}\left(0^{\prime },0^{\prime },0^{\prime }\right)\;=\;\ln2+0$ and the quantum correlation fluctuation $\Delta \delta \left(0,0,0,0^{\prime },0^{\prime },0^{\prime }\right)\;=\;\delta_{f}\left(0^{\prime },0^{\prime },0^{\prime }\right)-\delta_{i}\left(0,0,0\right)\;=\;0-\ln2$ in the configuration $\left(0,0,0,0^{\prime },0^{\prime },0^{\prime }\right)$ of $\left(m,a,b,m^{\prime },a^{\prime },b^{\prime }\right)$. Similarly, we have $\alpha \left(0,1,1,0^{\prime },0^{\prime },0^{\prime }\right)\;=\;\alpha_{i}\left(0,1,1\right)+\alpha_{f}\left(0^{\prime },0^{\prime },0^{\prime }\right)\;=\ln2+0$, $\Delta \delta \left(0,1,1,0^{\prime },0^{\prime },0^{\prime }\right)\;=\;\delta_{f}\left(0^{\prime },0^{\prime },0^{\prime }\right)-\delta_{i}\left(0,1,1\right)\;=\;0-\ln2$ in the configuration $\left(0,1,1,0^{\prime },0^{\prime },0^{\prime }\right)$.

The heat transferred from a heat bath to a two-level system is equal to $-\beta^{-1}\ln2$ during an isothermal process applied to each subsystem. This can be seen by the relation 〈Q〉=ΔE−W, where the energy change of the system is ΔE=0 and the work *W* done on the system $W=\beta^{-1}\ln2$, just like a particle becoming confined to only one half of two compartments in the final state. The total amount of heat transfer is then given by

(18) $$\begin{array}{c} \langle Q\rangle =-2\times \beta^{-1}\ln2. \end{array}$$

Moreover, the entropy change in the subsystem A(B)$\langle \Delta s_{A(B)}\rangle$ is computed by using the multi-indexed joint probability

(19) $$\begin{array}{cc} \langle \Delta s_{A(B)}\rangle & =p_{0,0,0,0^{\prime },0^{\prime },0^{\prime }}\left(-\ln p_{0^{\prime }}^{A(B)}+\ln p_{0}^{A(B)}\right)\\ & +p_{0,1,1,0^{\prime },0^{\prime },0^{\prime }}\left(-\ln p_{0^{\prime }}^{A(B)}+\ln p_{1}^{A(B)}\right)\\ \; & =\frac{1}{2}\times \left(-0+\ln\frac{1}{2}\right)+\frac{1}{2}\times \left(-0+\ln\frac{1}{2}\right)\\ \; & =-\ln2, \end{array}$$

where we used $\ln p_{0^{\prime }}^{A(B)}\;=\;0$, $\ln p_{0}^{A(B)}\;=\;\ln\frac{1}{2}$, and $\ln p_{1}^{A(B)}\;=\;\ln\frac{1}{2}$, since the initial and the final state are given by $\rho_{A(B)}^{i}\;=\;\frac{1}{2}{\left(|0\rangle \langle 0|+|1\rangle \langle 1|\right)}_{A(B)}$ and $\rho_{AB}^{f}\;=\;|0^{\prime }\rangle \langle 0^{\prime }{|}_{A}⊗|0^{\prime }\rangle \langle 0^{\prime }{|}_{B}$, respectively.

We then compute the average of quantum mutual information content −〈ΔI〉 in the inequality. Based on the definition of time-forward average, the mutual information is given by

(20) $$\begin{array}{cc} -\langle \Delta I\rangle & =\sum_{m,m^{\prime }}p_{m,0,0,m^{\prime },0^{\prime },0^{\prime }}\left(I_{0,0}-I_{0^{\prime },0^{\prime }}\right)\\ & +\sum_{m,m^{\prime }}p_{m,1,1,m^{\prime },0^{\prime },0^{\prime }}\left(I_{1,1}-I_{0^{\prime },0^{\prime }}\right)\\ \; & =p_{0,0,0,0^{\prime },0^{\prime },0^{\prime }}\left(I_{0,0}-I_{0^{\prime },0^{\prime }}\right)+p_{0,1,1,0^{\prime },0^{\prime },0^{\prime }}\left(I_{1,1}-I_{0^{\prime },0^{\prime }}\right)\\ \; & =\frac{1}{2}\left(\ln2-0\right)+\frac{1}{2}\left(\ln2-0\right)\\ \; & =\ln2. \end{array}$$

As a result, each term of the heat inequality (10) reads as

(21) $$\begin{array}{c} -2\ln2\;=\;-2\ln2+\ln2-\ln2, \end{array}$$

which confirms the saturation of the inequality. Note that the entire process is an irreversible process with the number of the non-zero configuration in the time-reversed process larger than that in the time forward process, which may be related to the absolute irreversibility [70]. In contrast, the usual second law of entropy production, without our term lnκ in (10), gives $\langle \sigma_{tot}\rangle =-\beta \langle Q\rangle +\langle \Delta s_{A}\rangle +\langle \Delta s_{B}\rangle -\langle \Delta I\rangle =2\ln2-\ln2-\ln2+\ln2>0$, where $\langle \sigma_{tot}\rangle$ represents the total entropy change of the system plus environment. Our inequality thus provides a useful tool to analyze an irreversible process of quantum bipartite system providing a tigheter bound with the quantum correlation factor κ.

### 3.3. Work Inequality

We now illustrate the merit of our derived inequality (14) in addressing work statistics during an irreversible process of quantum bipartite system. Let us consider a two qubit model where the time-dependent total Hamiltonian controlled by a parameter is given by H(λ(t)). The process is realized by slowly changing the parameter in which adiabatic approximation holds so that there is no transition between states. At the initial time, we assume that $[\rho_{i},H_{i}]\;=\;0$. Moreover, the state is in equilibrium $\rho_{AB}^{i}\;=\;\sum_{m}\frac{e^{-\beta E_{m}^{i}}}{Z_{AB}^{i}}\left|m\rangle \langle m\right|$ and the eigenbasis of the Hamiltonian corresponds to the Bell states $|\Psi^{+}\rangle ,|\Psi^{-}\rangle ,|\Phi^{+}\rangle ,|\Phi^{-}\rangle$, indexed as |m=0〉,|1〉,|2〉,|3〉 respectively. The Bell states here are $|\Psi^{\pm }\rangle \;=\;1/\sqrt{2}\left(|00\rangle \pm |11\rangle \right)$ and $|\Phi^{\pm }\rangle \;=\;1/\sqrt{2}\left(|01\rangle \pm |01\rangle \right)$. On the other hand, the final state $\rho_{f}$ is assumed to satisfy $[\Pi_{m},\Pi_{a,b}]=0$ so that the final states are product states as $|m^{\prime }\rangle \;=\;|a^{\prime },b^{\prime }\rangle \;=\;|a^{\prime }\rangle |b^{\prime }\rangle$ for all the indices.

**Figure 2:**
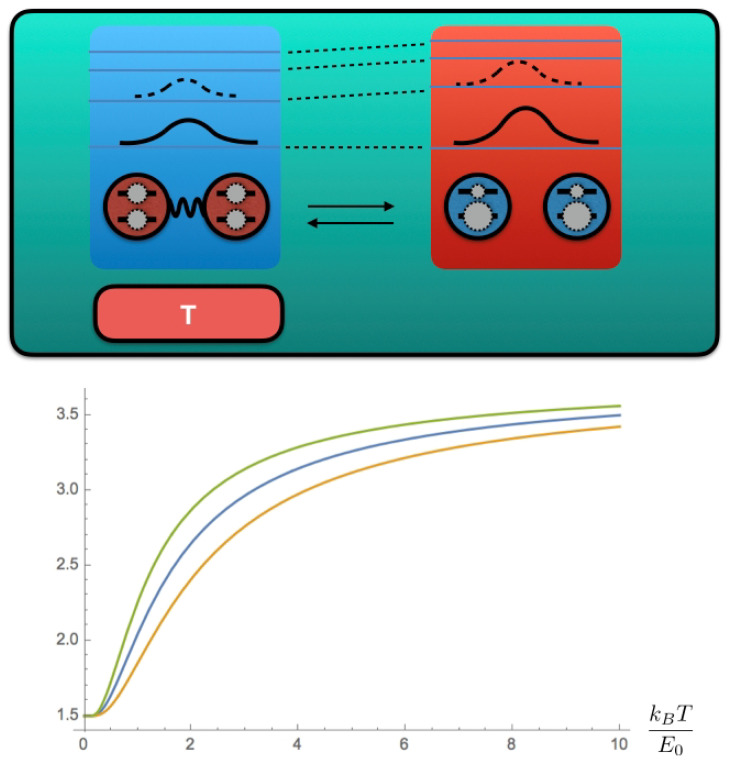
Top: schematic of a quantum adiabatic process for a quantum correlated bipartite system. Bottom: thermodynamic quantities $\langle w\rangle$ (Green), $\Delta f_{qc}-\beta^{-1}\ln\kappa$ (Blue) and $\Delta F_{AB}$ (Orange).

We may readily see that the observation of quantum fluctuations in a time-reversed protocol makes it possible to improve predictions on the thermodynamic variables through our example. To show it, we numerically show the relationship between $\langle \Delta f_{qc}\rangle -\beta^{-1}\ln\kappa$ (blue curve) and $\Delta F_{AB}$ (usual bound for work, orange curve) in Figure 2. In a regime that $\langle \Delta f_{qc}\rangle -\beta^{-1}\ln\kappa \geq \Delta F_{AB}$, our inequality provides a tighter bound so that the work 〈w〉 (green curve) is compared with other quantities as expected to be $\langle w\rangle \geq \langle \Delta f_{qc}\rangle -\beta^{-1}\ln\kappa \;\geq \;\Delta F_{AB}$.

## 4. Summary

We have obtained a fluctuation theorem in Equation (4) for an open bipartite quantum system that explicitly incorporates genuinely quantum fluctuations due to non-commutativity. In particular, the term κ represents quantum nature of thermodynamics by using the quantities α and δ defined around Equation (6), which manifest quantum correlation. Our FT specifically looks into the quantum correlation in time-reversed process thereby providing not only a novel perspective but also useful thermodynamic inequalities. We have illustrated our inequalities on heat transfer and work through two examples of quantum correlated systems, one under an isothermal process and the other under an adiabatic process. These demonstrate the merit of our approach yielding tighter bounds for thermodynamically relevant quantities.

One of the main problems in quantum thermodynamics is to understand how quantum principles may modify the known results in classical thermodynamics. It is crucial to identify the role played by quantum correlation in the emerging non-equilibrium thermodynamics, which may shed light into the question, e.g., how the environmental interaction may affect the performance of quantum systems in quantum information processing. Our work has here focused on the inter-correlation between quantum systems of interest, but it will be a meaningful extension to include the effect of system-reservoir correlation in understanding the emergent quantum dynamics. For instance, a recent work in [71] developed a quantum master equation that overcomes the pathological problems, like non-positivity of populations in case of the Redfield or the Lindblad equation. This approach can be adopted to address the effect of system-reservoir correlation in the low-orders of environmental coupling. We hope our work here could stimulate further useful works along this line particularly in view of the merit by formulating thermodynamic statistics in time-reversed process.

## Data Availability

The data presented in this study are available on request from the corresponding author. The data are not publicly available since the data related to the Figure 2 can be readily produced through the explanation in main text.

## Appendix A. Derivation of Equation (6)

Defining $\alpha_{i(f)}\;=\;-\ln{\left|\langle m^{\left(\prime \right)}|a^{\left(\prime \right)},b^{\left(\prime \right)}\rangle \right|}^{2}\;=\;\ln p_{m(m^{\prime })}-\ln p_{m\left(m^{\prime }\right),a\left(a^{\prime }\right),b\left(b^{\prime }\right)}$, we obtain

(A1) $$\begin{array}{ccc} & & p_{m,a,b,m^{\prime },a^{\prime },b^{\prime };r,r^{\prime }}\\ & = & |\langle m^{\prime },r^{\prime }{\left|U|m,r\rangle \right|}^{2}p_{m}p_{r}e^{-\alpha_{i}}e^{-\alpha_{f}}\\ & = & \left|\langle m,r\right|U^{†}|m^{\prime },r^{\prime }{\rangle |}^{2}{\tilde{p}}_{m^{\prime }}{\tilde{p}}_{r^{\prime }}\frac{p_{m}p_{r}}{{\tilde{p}}_{m^{\prime }}{\tilde{p}}_{r^{\prime }}}e^{-\alpha_{i}}e^{-\alpha_{f}}\\ & = & \left|\langle m,r\right|\Theta^{†}\Theta U^{†}\Theta^{†}\Theta |m^{\prime },r^{\prime }{\rangle |}^{2}{\tilde{p}}_{m^{\prime }}{\tilde{p}}_{r^{\prime }}\frac{p_{m}p_{r}}{{\tilde{p}}_{m^{\prime }}{\tilde{p}}_{r^{\prime }}}e^{-\tilde{\alpha_{i}}}e^{-\tilde{\alpha_{f}}}\\ & = & |\langle \tilde{m},\tilde{r}|\tilde{U}|\tilde{m^{\prime }},\tilde{r^{\prime }}{\rangle |}^{2}e^{-{\tilde{\alpha }}_{f}}e^{-{\tilde{\alpha }}_{i}}{\tilde{p}}_{m^{\prime }}{\tilde{p}}_{r^{\prime }}\frac{p_{m}p_{r}}{{\tilde{p}}_{m^{\prime }}{\tilde{p}}_{r^{\prime }}}\\ & = & {\tilde{p}}_{m^{\prime },a^{\prime },b^{\prime },m,a,b;r^{\prime },r}\frac{p_{a}}{{\tilde{p}}_{a^{\prime }}}\frac{p_{b}}{{\tilde{p}}_{b^{\prime }}}\frac{p_{r}}{{\tilde{p}}_{r^{\prime }}}\left[\frac{p_{a,b}}{p_{a}p_{b}}\frac{{\tilde{p}}_{a^{\prime }}{\tilde{p}}_{b^{\prime }}}{{\tilde{p}}_{a^{\prime },b^{\prime }}}\right]\left[\frac{p_{m}}{p_{a,b}}\frac{{\tilde{p}}_{a^{\prime },b^{\prime }}}{{\tilde{p}}_{m^{\prime }}}\right]\\ & = & {\tilde{p}}_{m^{\prime },a^{\prime },b^{\prime },m,a,b;r^{\prime },r}e^{\Delta s_{A}}e^{\Delta s_{B}}e^{\Delta s_{R}}e^{-\Delta I}e^{-\Delta \delta }, \end{array}$$

where $\Delta s_{A}:=\ln\frac{p_{a}}{{\tilde{p}}_{a^{\prime }}},\Delta s_{B}:=\ln\frac{p_{b}}{{\tilde{p}}_{b^{\prime }}},\Delta s_{R}:=\ln\frac{p_{r}}{{\tilde{p}}_{r^{\prime }}},\Delta I:=I_{f}-I_{i}\;=\;\ln\frac{{\tilde{p}}_{a^{\prime },b^{\prime }}}{{\tilde{p}}_{a^{\prime }}{\tilde{p}}_{b^{\prime }}}-\ln\frac{p_{a,b}}{p_{a}p_{b}}$, and $\Delta \delta :=\delta_{f}-\delta_{i}\;=\;\ln\frac{{\tilde{p}}_{m^{\prime }}}{{\tilde{p}}_{a^{\prime },b^{\prime }}}-\ln\frac{p_{m}}{p_{a,b}}$. We have also used the relation $e^{-\alpha_{i}}\;=\;e^{-{\tilde{\alpha }}_{f}}$ since ${\left|\langle m|a,b\rangle \right|}^{2}=\left|\langle m\right|\Theta^{†}{\Theta |a,b\rangle |}^{2}={\left|\langle \tilde{m}|\tilde{a},\tilde{b}\rangle \right|}^{2}$, and similarly $e^{-\alpha_{f}}\;=\;e^{-{\tilde{\alpha }}_{i}}$.

## Appendix B. Proof of Equation (11)

We prove Equation (11) as follows.

(A2) $$\begin{array}{ccc} & & p_{m,a,b,m^{\prime },a^{\prime },b^{\prime }}\\ & = & |\langle m^{\prime }{\left|U|m\rangle \right|}^{2}{\left|\langle m|a,b\rangle \right|}^{2}{\left|\langle m^{\prime }|a^{\prime },b^{\prime }\rangle \right|}^{2}p_{m}\\ & = & \left|\langle m\right|U^{†}|m^{\prime }{\rangle |}^{2}{\left|\langle m|a,b\rangle \right|}^{2}{\left|\langle m^{\prime }|a^{\prime },b^{\prime }\rangle \right|}^{2}{\tilde{p}}_{m^{\prime }}\frac{p_{m}}{{\tilde{p}}_{m^{\prime }}}\\ & = & {\tilde{p}}_{m^{\prime },a^{\prime },b^{\prime },m,a,b}\left[\frac{p_{a,b}}{{\tilde{p}}_{a^{\prime },b^{\prime }}}\right]\left[\frac{p_{m}}{p_{a,b}}\frac{{\tilde{p}}_{a^{\prime },b^{\prime }}}{{\tilde{p}}_{m^{\prime }}}\right]\\ & = & {\tilde{p}}_{m^{\prime },a^{\prime },b^{\prime },m,a,b}e^{\beta w-\beta [\left(E_{m^{\prime }}^{f}-E_{m}^{i}\right)-\beta^{-1}\ln\frac{p_{a.b}}{p_{a^{\prime },b^{\prime }}}]-\Delta \delta }\\ & = & {\tilde{p}}_{m^{\prime },a^{\prime },b^{\prime },m,a,b}e^{\beta (w-\Delta f_{qc})-\Delta \delta }, \end{array}$$

with the quantum fluctuation $\delta_{i(f)}\;=\;\ln p_{m(m^{\prime })}-\ln p_{a\left(a^{\prime }\right),b\left(b^{\prime }\right)}$. That is, we obtain

(A3) $$p_{m,a,b,m^{\prime },a^{\prime },b^{\prime };r,r^{\prime }}e^{-\beta (w-\Delta f_{qc})}\;=\;{\tilde{p}}_{m^{\prime },a^{\prime },b^{\prime },m,a,b;r^{\prime },r}e^{-\Delta \delta },$$

and Equation (11) is derived in the same way as done with Equation (4), with $\kappa \;=\sum_{m,m^{\prime }}{\tilde{p}}_{m^{\prime },m}\sum_{a,a^{\prime },b,b^{\prime }}e^{-[\alpha +\Delta \delta ]}$.

## Appendix C. Calculation of Relevant Quantities for the Example in Section 3.1

### Appendix C.1. Time-Forward Process

Let us extend the isothermal process in Section 3.1 to each equilibrium subsystem of a maximally entangled bipartite system [69]. The joint system is initially in the state $\rho_{AB}^{i}⊗\rho_{R}^{A}⊗\rho_{R}^{B}\;=\;\sum_{m=0}^{3}p_{m}\left|m\rangle \langle m\right|⊗\sum_{r}e^{-\beta E_{r}^{R}}/Z_{\beta }\left|r\rangle \langle r\right|⊗\sum_{r^{\prime }}e^{-\beta E_{r^{\prime }}^{R}}/Z_{\beta }|r^{\prime }\rangle \langle r^{\prime }|$ in the Bell-state basis given by

(A4) $$\begin{array}{cc} |0\rangle & =\frac{1}{\sqrt{2}}\left({|0\rangle }_{A}⊗{|0\rangle }_{B}+{|1\rangle }_{A}⊗{|1\rangle }_{B}\right),\\ |1\rangle & =\frac{1}{\sqrt{2}}\left({|0\rangle }_{A}⊗{|0\rangle }_{B}-{|1\rangle }_{A}⊗{|1\rangle }_{B}\right),\\ |2\rangle & =\frac{1}{\sqrt{2}}\left({|0\rangle }_{A}⊗{|1\rangle }_{B}+{|1\rangle }_{A}⊗{|0\rangle }_{B}\right),\\ |3\rangle & =\frac{1}{\sqrt{2}}\left({|0\rangle }_{A}⊗{|1\rangle }_{B}-{|1\rangle }_{A}⊗{|1\rangle }_{B}\right). \end{array}$$

After applying the aforementioned isothermal process to the bipartite system, the initial state |0〉 is changed to the final state $\rho_{AB}^{f}=|0^{\prime }\rangle \langle 0^{\prime }\left|\;=\;\right|0^{\prime }\rangle \langle 0^{\prime }{|}_{A}⊗|0^{\prime }\rangle \langle 0^{\prime }{|}_{B}$ in the eigenbases

(A5) $$\begin{array}{cc} |0^{\prime }\rangle_{AB} & =|0^{\prime }\rangle_{A}⊗{|0^{\prime }\rangle }_{B},\\ |1^{\prime }\rangle_{AB} & =|0^{\prime }\rangle_{A}⊗{|1^{\prime }\rangle }_{B},\\ |2^{\prime }\rangle_{AB} & =|1^{\prime }\rangle_{A}⊗{|0^{\prime }\rangle }_{B},\\ |3^{\prime }\rangle_{AB} & =|1^{\prime }\rangle_{A}⊗{|1^{\prime }\rangle }_{B}. \end{array}$$

In this process, we evaluate the multi-indexed joint probabilities and the related contents as follows.

(1) The marginal probabilities $p_{m}$ and $p_{m^{\prime }}$ at the initial and the final time are

(A6) $$\begin{array}{cc} p_{0} & =1,\;p_{1,2,3}=0\\ p_{0^{\prime }} & \;=\;1,\;p_{1^{\prime },2^{\prime },3^{\prime }}=0 \end{array}$$

(2) The conditional probabilities ${\left|\langle m|a,b\rangle \right|}^{2}$ at the initial time are given by

(A7) $$\begin{array}{cc} {\left|\langle 0|0,0\rangle \right|}^{2} & ={\left|\langle 0|1,1\rangle \right|}^{2}\;=\;\frac{1}{2}{,|\langle 0|0,1\rangle |}^{2}\;=\;{\left|\langle 0|1,0\rangle \right|}^{2}=0;\\ {\left|\langle 1|0,0\rangle \right|}^{2} & ={\left|\langle 1|1,1\rangle \right|}^{2}\;=\;\frac{1}{2}{,|\langle 1|0,1\rangle |}^{2}\;=\;{\left|\langle 1|1,0\rangle \right|}^{2}\;=\;0;\\ {\left|\langle 2|0,0\rangle \right|}^{2} & ={\left|\langle 2|1,1\rangle \right|}^{2}\;={\;0,|\langle 2|0,1\rangle |}^{2}\;=\;{\left|\langle 2|1,0\rangle \right|}^{2}\;=\;\frac{1}{2};\\ {\left|\langle 3|0,0\rangle \right|}^{2} & ={\left|\langle 3|1,1\rangle \right|}^{2}\;={\;0,|\langle 3|0,1\rangle |}^{2}\;=\;{\left|\langle 3|1,0\rangle \right|}^{2}\;=\;\frac{1}{2}, \end{array}$$

and those at the final time are

(A8) $$\begin{array}{c} |\langle 0^{\prime }|0^{\prime },0^{\prime }\rangle {|}^{2}\;=\;1,|\langle 0^{\prime }|0^{\prime },1^{\prime }\rangle {|}^{2}\;=\;|\langle 0^{\prime }|1^{\prime },0^{\prime }\rangle {|}^{2}\;=\;{\left|\langle 0^{\prime }|1^{\prime },1^{\prime }\rangle \right|}^{2}\;=\;0;\\ |\langle 1^{\prime }|0^{\prime },1^{\prime }\rangle {|}^{2}\;=\;1,|\langle 1^{\prime }|0^{\prime },0^{\prime }\rangle {|}^{2}\;=\;|\langle 1^{\prime }|1^{\prime },0^{\prime }\rangle {|}^{2}\;=\;{\left|\langle 1^{\prime }|1^{\prime },1^{\prime }\rangle \right|}^{2}\;=\;0^{\prime };\\ |\langle 2^{\prime }|1^{\prime },0^{\prime }\rangle {|}^{2}\;=\;1,|\langle 2^{\prime }|0^{\prime },0^{\prime }\rangle {|}^{2}\;=\;|\langle 2^{\prime }|0^{\prime },1^{\prime }\rangle {|}^{2}\;=\;{\left|\langle 2^{\prime }|1^{\prime },1^{\prime }\rangle \right|}^{2}\;=\;0^{\prime };\\ |\langle 3^{\prime }|1^{\prime },1^{\prime }\rangle {|}^{2}\;=\;1,|\langle 3^{\prime }|0^{\prime },0^{\prime }\rangle {|}^{2}\;=\;|\langle 3^{\prime }|0^{\prime },1^{\prime }\rangle {|}^{2}\;=\;{\left|\langle 3^{\prime }|1^{\prime },0^{\prime }\rangle \right|}^{2}\;=\;0. \end{array}$$

Note that these values determine the factor $e^{-\alpha }$ which describes quantum fluctuations arising from the incompatibility between the joint state and the local states. Constructing the multi-indexed joint probabilities are given by using the factor $e^{-\alpha }$, as shown below.

(3) The time-forward multi-indexed joint probability given by

$$p_{m,m^{\prime };r,r^{\prime }}\;=\;|\langle m^{\prime },r^{\prime }{\left|U|m,r\rangle \right|}^{2}p_{m}p_{r}$$

may be reduced to a joint probability for an open quantum bipartite system as

$$p_{m,m^{\prime }}\;=\;\sum_{r,r^{\prime }}p_{m,m^{\prime };r,r^{\prime }}\;=\;\sum_{r,r^{\prime }}|\langle m^{\prime }|M_{r,r^{\prime }}{\left|m\rangle \right|}^{2}p_{m},$$

where $M_{r,r^{\prime }}\;=\;\langle r^{\prime }|U|r\rangle \sqrt{p_{r}}$. In our example, the allowed transition between |m〉 and $|m^{\prime }\rangle$ is a single transition between |0〉 and $|0^{\prime }\rangle$ so that $\sum_{r,r^{\prime }}|\langle m^{\prime }|M_{r,r^{\prime }}{\left|m\rangle \right|}^{2}p_{m}=1$ for m=0 and $m^{\prime }=0^{\prime }$ and otherwise $\sum_{r,r^{\prime }}|\langle m^{\prime }|M_{r,r^{\prime }}{\left|m\rangle \right|}^{2}p_{m}=0$. We then obtain

$$p_{0,0^{\prime }}\;=\;1,\;\mathrm{otherwise}\;p_{m,m^{\prime }}\;=\;0.$$

Based on the above quantities, we are able to compute different types of joint probabilities $p_{m,a,b}$ and $p_{m,a,b,m^{\prime },a^{\prime },b^{\prime }}$ by using the factor $e^{-\alpha }$. Since $p_{m,a,b}\;=\;e^{-\alpha }p_{m}$ with $e^{-\alpha }\;=\;e^{-[-\ln|\langle m|a,b\rangle {|}^{2}]}$, the probabilities at $t_{i}$ and $t_{f}$ are respectively given by

(A9) $$p_{0,0,0}\;=\;p_{0,1,1}\;=\;\frac{1}{2}\;\mathrm{at}\;t_{i}\;\mathrm{and}\;\;p_{0^{\prime },0^{\prime },0^{\prime }}\;=\;1\;\mathrm{at}\;t_{f}.$$

Otherwise, all the others such as $p_{1,0,0}$, $p_{2,0,1}$ are zero. Then, the full multi-indexed joint probability $p_{m,a,b,m^{\prime },a^{\prime },b^{\prime }}$ is readily obtained by $p_{m,a,b,m^{\prime },a^{\prime },b^{\prime }}\;=\;p_{m,m^{\prime }}e^{-\alpha_{i}}e^{-\alpha_{f}}$, with the non-zero values given as

(A10) $$p_{0,0,0,0^{\prime },0^{\prime },0^{\prime }}\;=\;p_{0,1,1,0^{\prime },0^{\prime },0^{\prime }}\;=\;\frac{1}{2}.$$

### Appendix C.2. Time-Reversed Process

Similarly, we assess the joint probabilities in a time-reversed protocol by isothermally changing the energy gap from Δ to 0 in a time-reversed manner. During this process, the initial density operator ${\tilde{\rho }}_{AB}^{i}\;=\;|0^{\prime }\rangle \langle 0^{\prime }{|}^{AB}$ is changed to the density operator at the final time ${\tilde{\rho }}_{AB}^{f}\;=\;\frac{1}{2}{\left(|0\rangle \langle 0|+|1\rangle \langle 1|\right)}_{A}⊗\frac{1}{2}{\left(|0\rangle \langle 0|+|1\rangle \langle 1|\right)}_{B}$. We obtain the entropy and the information content for the time-reversed protocol as

(1${}^{\prime }$) The marginal probabilities ${\tilde{p}}_{m}$ and ${\tilde{p}}_{m^{\prime }}$ at the initial and the final time in the reversed protocol is

(A11) $$\begin{array}{c} {\tilde{p}}_{m}\;=\;1\;\mathrm{for}\;m=0\;\mathrm{and}\;{\tilde{p}}_{m^{\prime }}\;=\;1/4\;\mathrm{for}\;m\;=\;0,1,2,3. \end{array}$$

(2${}^{\prime }$) One may find the time-reversed joint probabilities for the transitions from the initial state $|0^{\prime }\rangle$ to the final states |0〉,|1〉,|2〉,|3〉 as

$${\tilde{p}}_{0^{\prime },0}\;=\;{\tilde{p}}_{0^{\prime },1}\;=\;{\tilde{p}}_{0^{\prime },2}\;=\;{\tilde{p}}_{0^{\prime },3}\;=\;\frac{1}{4},$$

and all the others are zero.

(3${}^{\prime }$) By taking the above contents, the reduced joint probability ${\tilde{p}}_{m^{\prime },a^{\prime },b^{\prime }}$ at $t_{f}$ and $t_{i}$ are respectively given by

(A12) $$\begin{array}{cc} & {\tilde{p}}_{0^{\prime },0^{\prime },0^{\prime }}\;=\;1\;\mathrm{at}\;t_{f},\\ & {\tilde{p}}_{0,0,0}\;=\;{\tilde{p}}_{1,0,1}\;=\;{\tilde{p}}_{2,1,0}\;=\;{\tilde{p}}_{3,1,1}=\frac{1}{4}\;\mathrm{at}\;t_{i}. \end{array}$$

Otherwise, all the other probabilities of ${\tilde{p}}_{m^{\prime },a^{\prime },b^{\prime }}$ and ${\tilde{p}}_{m,a,b}$ are zero. In addition, the non-zero configuration of the multi-indexed time-reversed joint probability ${\tilde{p}}_{m^{\prime },a^{\prime },b^{\prime },m,a,b}\;={\tilde{p}}_{m^{\prime },m}e^{-\alpha_{f}}e^{-\alpha_{i}}$ is given as ${\tilde{p}}_{m^{\prime },a^{\prime },b^{\prime },m,a,b}\;=\;\frac{1}{4}$ for the transitions $\left(0^{\prime },0^{\prime },0^{\prime },0,0,0\right)$,$\left(0^{\prime },0^{\prime },0^{\prime },1,0,1\right)$, $\left(0^{\prime },0^{\prime },0^{\prime },2,1,0\right)$, and $\left(0^{\prime },0^{\prime },0^{\prime },3,1,1\right)$.
